# Regulation and Role of Arabidopsis CUL4-DDB1A-DDB2 in Maintaining Genome Integrity upon UV Stress

**DOI:** 10.1371/journal.pgen.1000093

**Published:** 2008-06-13

**Authors:** Jean Molinier, Esther Lechner, Eva Dumbliauskas, Pascal Genschik

**Affiliations:** Institut de Biologie Moléculaire des Plantes du CNRS (UPR2357), conventionné avec l'Université Louis Pasteur, Strasbourg, France; The University of North Carolina at Chapel Hill, United States of America

## Abstract

Plants use the energy in sunlight for photosynthesis, but as a consequence are exposed to the toxic effect of UV radiation especially on DNA. The UV-induced lesions on DNA affect both transcription and replication and can also have mutagenic consequences. Here we investigated the regulation and the function of the recently described CUL4-DDB1-DDB2 E3 ligase in the maintenance of genome integrity upon UV-stress using the model plant Arabidopsis. Physiological, biochemical, and genetic evidences indicate that this protein complex is involved in global genome repair (GGR) of UV-induced DNA lesions. Moreover, we provide evidences for crosstalks between GGR, the plant-specific photo reactivation pathway and the RAD1-RAD10 endonucleases upon UV exposure. Finally, we report that DDB2 degradation upon UV stress depends not only on CUL4, but also on the checkpoint protein kinase Ataxia telangiectasia and Rad3-related (ATR). Interestingly, we found that DDB1A shuttles from the cytoplasm to the nucleus in an ATR-dependent manner, highlighting an upstream level of control and a novel mechanism of regulation of this E3 ligase.

## Author Summary

Recent research revealed strong links between Cullin4 (CUL4)–based cullin-RING ubiquitin ligases (CRLs) and chromatin biology, including DNA replication and DNA repair. During Nucleotide Excision Repair (NER), CUL4 together with DDB1 (DNA Damage Binding protein 1) ubiquitylate an increasingly large number of substrates, including components of the NER machinery as well as various histone proteins. In contrast to mammals, plants have an efficient DNA repair pathway, mediated by photolyases that reverse UV lesions in presence of visible light, without DNA excision. Thus, it is believed that in aerial plant tissues this DNA repair pathway is predominant. In the present work we used the model plant Arabidopsis to investigate the role of CUL4-DDB1A^DDB2^ in global genome repair at the level of a whole organism. Using a genetic approach, we highlighted the existence of cooperative roles of different DNA repair processes that all together contribute to maintain genome integrity upon exposure to UV. Moreover, we report that DDB2 turnover not only depends on CUL4-DDB1A, but also on the ATR checkpoint protein kinase. Strikingly, we demonstrated that Arabidopsis DDB1A shuttles from the cytoplasm to the nucleus after UV stress in an ATR-dependent manner, illustrating a novel level of regulation of this class of CRL.

## Introduction

Plants are subjected to many different environmental stresses due to their sessile life styles. As obligate prototroph they need to take benefit of the sunlight containing ultra-violets (UV) for photosynthesis but concomitantly, have to prevent UV irradiation from inducing irreversible damage especially on DNA. Genetic changes that occur in somatic plant cells can potentially be passed to the progeny through meiosis since plants lack a permanent germ line and produce gametes from somatic lineages [1]. In order to avoid dramatic genetic alterations efficient DNA repair pathways must be activated in addition to physiological adaptation like biosynthesis of UV absorbing compounds. UVs predominantly induce photochemical lesions such as cyclobutane pyrimidine dimers (CPDs) and (6-4)-photoproducts [2]. These bulky DNA lesions are repaired by a simple and error free mechanism: photo reactivation, which is present in prokaryotes and most eukaryotes, with the exception of mammals [3]. In the model plant *Arabidopsis thaliana*, the *UVR2* gene encodes a photolyase (PHR1) that acts only on CPDs [4],[5], whereas the *UVR3* gene encodes a photolyase specific for 6-4 photoproducts [6].

In addition to this direct repair process, most organisms also have a sophisticated general repair mechanism, such as the Nucleotide Excision Repair (NER) [7]. The NER process contributes to global genome repair and consists of 5 steps: recognition of the DNA damage, opening of the pre-incision complex, dual incision of the lesion, DNA synthesis followed by the ligation of the newly synthesised DNA strand. Two proteins, DDB1 and DDB2 (DNA Damage Binding protein 1 and 2), were initially isolated as part of a heterodimeric protein complex that recognises UV-induced DNA lesions [8]. Although still under debate, these proteins seem to play an early function during NER. Recent finding have shown that DDB1 and DDB2, at least in mammals, form an E3 ubiquitin ligase together with Cullin 4 (CUL4) and RBX1 [9]. Thus, CUL4 binds RBX1 to recruit a specific E2 ubiquitin conjugating enzyme and also binds DDB1, an adaptor protein, which itself associates with the predicted substrate receptor DDB2. However, the ubiquitylation events triggered by this E3 ligase during NER seem complex and are not yet fully understood. Thus in mammals, both the *Xeroderma pigmentosum* complementation group C (XPC) and the receptor protein itself, DDB2, are ubiquitylated after UV irradiation [10], [11]–[12], [13]–[14]. However the fate of these proteins are different; whereas polyubiquitylated XPC is not degraded and exhibits enhanced binding activity to damaged DNA, polyubiquitylated DDB2 is destroyed by the proteasome. Thus it is believed that DDB2 facilitates and/or stabilizes the binding of XPC to UV-induced photoproducts [15], but its turnover is crucial to allow the NER process to occur efficiently. Therefore recognition of UV lesions at the initial step of NER is critical and should be tightly controlled to allow efficient and rapid DNA repair. Hence mutations in human *DDB2* gene cause genetic complementation group XPE phenotype characterized by hypersensitivity to sunlight and a predisposition to skin cancer [16]. Interestingly, recent data also indicate a role of CUL4-DDB1-DDB2 E3 ligase in histone H3 and H4 ubiquitylation during UV stress [17]. This work points to an interesting model in which histone ubiquitylation around the lesions may cause the eviction of nucleosomes and thus expose the damaged DNA to the repair machinery. A function of CUL4A-DDB1^DDB2^ was also proposed for histone H2A monoubiquitylation during UV irradiation [18]. Finally, DDB2 may also act in NER in a chromatin-independent and less direct manner, by controlling the stability of P53 [19].

In spite of these advances, the control and the dynamics of the CUL4-DDB1-DDB2 complex during the recognition step of photoproducts is still not well understood. Moreover, the cooperation between the different DNA repair pathways in the repair of highly mutagenic UV-induced DNA lesions remains poorly characterized and much of our current knowledge comes from data obtained with different cell types and not at the level of a complex organism. In the present work we used the model plant *Arabidopsis thaliana,* which has to deal with the benefit of UV light and the harmful effects of photoproducts on DNA during development. Here we provide evidences for a CUL4-DDB1A^DDB2^ protein complex *in planta* and for its function in the global genome repair pathway (GGR). Using a genetic approach, we demonstrate a synergism between GGR, the plant specific photo reactivation pathway and the RAD1-RAD10 endonucleases upon UV exposure. These results support the existence of different interconnections between DNA repair processes that all together contribute to maintain genome integrity. Moreover, we report that DDB2's turnover depends on the CUL4-DDB1A E3 ligase and the ATR kinase. Strikingly, we demonstrated that Arabidopsis DDB1A shuttles from the cytoplasm to the nucleus in an ATR-dependent manner, highlighting an upstream control and a novel level of regulation for this CUL4-based E3 ligase.

## Results

### Molecular Characterisation of Arabidopsis *DDB1A* and *DDB2* Insertion Lines

**Figure 1:**
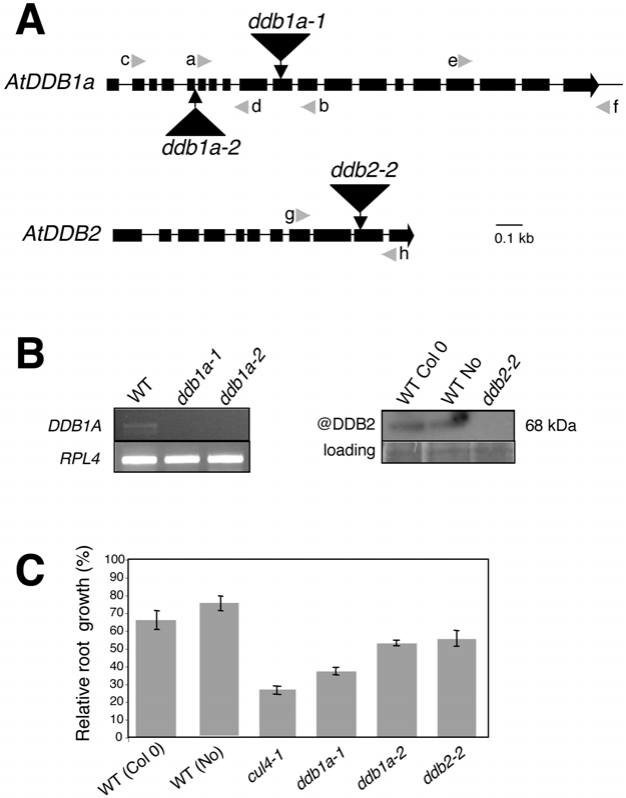
*cul4*, *ddb1a* and *ddb2* insertion mutant lines exhibit hypersensitivity to UV-C. (A) Genomic organization of the *DDB1A* and *DDB2* loci. T-DNAs are inserted in the 10^th^ exon (black boxes) in the *ddb1a-1* and in the 5^th^ intron (black line) in *ddb1a-2* mutant plants. Primers a–b, c–d and LBb1 were used for genotyping *ddb1a-1* and *ddb1a-2,* respectively. In the *ddb2-2*, the transposon is inserted in the 10^th^ exon (black boxes). Primers h–g and Ds5-2a were used for genotyping (see supplemental material for details). (B) Molecular characterisation of DDB1A- and DDB2-mutant plants. RT-PCR analyses were performed for *DDB1A* expression in WT and the insertional mutants *ddb1a-1* and *ddb1a-2* using the specific e–f primers. *RPL4* primers were used as a control (See supplemental material for details). Immuno-detection of DDB2 protein in two different WT *Arabidopsis thaliana* ecotypes (Columbia: Col0 and Nossen: No) and in the *ddb2-2* insertional mutant. Coomassie blue staining was used as loading control. (C) Root-growth assay. One-week-old mutant (*cul4-1*, *ddb1a-1*, *ddb1a-2*, *ddb2-2*), and WT control plants were exposed to 600 J/m^2^ of UV-C. Root growth was measured 24h following irradiation. Root growth was calculated relative to the corresponding untreated plants (±SEM). Eight plants per replicate were used and experiments were performed in triplicates. For all mutants p<0.05, compared to WT plants (both ecotypes).

In order to define the role of Arabidopsis CUL4, DDB1A and DDB2 in DNA repair, a genetic approach was conducted. Two Arabidopsis *DDB1A* (*ddb1a-1* and *ddb1a-2*) and one *DDB2* (*ddb2-2*) insertion lines were identified using the web-assisted program: http://signal.salk.edu/cgi-bin/tdnaexpress. The Arabidopsis *CUL4* insertion line used in this study (*cul4-1*) is described in [20]. We used this hypomorphic mutant, which exhibits a strong reduction of *CUL4* expression, instead of a null mutant that is lethal in Arabidopsis (unpublished). In lines *ddb1a-1* and *ddb1a-2* the T-DNAs are integrated in the 10^th^ exon and in the 5^th^ intron, respectively (Figure 1A). For both tagged lines, Arabidopsis plants homozygous for the T-DNA insertions did not exhibit significant phenotypical differences compared to wild-type (WT) plants under standard growth conditions. Using RT-PCR *DDB1A* transcripts remain undetectable in *ddb1a-1* and *ddb1a-2* mutant plants (Figure 1B) indicating that both mutant lines are defective for *DDB1A* expression. For DDB2, which is encoded by a single gene in Arabidopsis, we raised antibodies against the protein for its immunodetection in different mutant lines (see experimental procedures for details). One T-DNA insertion mutant for *AtUV-DDB2* (here referred as *ddb2-1*) was recently reported [21], but this mutant still expresses low amount of DDB2 protein (not shown). For this reason, we chose another mutant line, *ddb2-2* (Nossen ecotype), in which a transposon is integrated in the 10^th^ exon (Figure 1A). No signal on western blot is detectable in *ddb2-2* (Figure 1B), suggesting a loss-of-function mutation. Similarly to *ddb1a* mutants, plants homozygous for this insertion did not exhibit significant phenotypic differences compared to the wild type Arabidopsis plants grown under standard conditions. It is noteworthy that *DDB2* expression level in both Col0 and Nossen WT Arabidopsis ecotypes is low (Figure 1B), which is in agreement with the signal intensity of several different microarray experiments reflecting the transcript level [22].

### *CUL4*, *DDB1A,* and *DDB2* Defective Plants Are UV-C Hypersensitive

To test whether Arabidopsis *CUL4*, *DDB1A* and *DDB2* are involved in the repair of UV-induced DNA lesions, root growth inhibition experiments were performed. In plants, roots are the material of choice to measure the defect in UV tolerance, because they lack UV-screen compounds, which protect cells from UV light. *cul4*, *ddb1a* and *ddb2* defective mutant plants were grown vertically on the agar surface and roots were irradiated with 600 J/m^2^ of UV-C. Relative root growth (root length treated/ root length untreated) in % was determined 24h following the UV-C treatment. Root growth was reduced to 28, 37, 50 and 53% for *cul4-1*, *ddb1a-1*, *ddb1a-2* and *ddb2-2*, respectively, relative to that of WT control plants (Figure 1C). Therefore all insertion lines exhibit a significant root growth inhibition upon exposure to UV-C compared to WT Arabidopsis plants. Interestingly, both Arabidopsis DDB1A and DDB2 over expressing plants exhibit an enhanced UV-C tolerance compared to WT plants (Figure S1A) suggesting that both proteins, as effectors of the excision repair pathway, are limiting factors in this process.

Cisplatin is another DNA damaging agent that induces bulky DNA lesions. Similarly to UV-C stress, *cul4-1*, *ddb1a-2* and *ddb2-2* exhibit a significant growth reduction compared to WT control plants, as measured by their relative weight (Figure S1B). However, we did not observe such a difference in sensitivity upon exposure of the mutant lines to hydrogen peroxide (H_2_O_2_; Figure S1C). This lack of sensitivity is in agreement with the fact that H_2_O_2_ induced DNA lesions are not predominantly repaired by the NER pathway. Taken together these data suggest that *CUL4*-, *DDB1A*- and *DDB2*-deficient plants are affected in the tolerance to induced bulky DNA damage and this may reflect a defect in DNA repair.

### *CUL4* and *DDB2* Defective Plants Are Impaired in Synthesis-Dependent Repair of UV-Induced DNA Lesions

**Figure 2:**
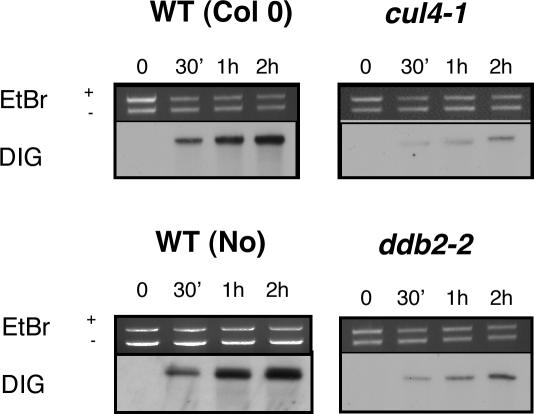
*In vitro* synthesis DNA repair assays of UV-C damaged plasmid. Cell extracts (20 µg) from WT (Col/Nossen), *cul4-1* and *ddb2-2* plants were incubated with UV-C damaged (UV-C treated pGEX: +UV-C) and control (untreated pBKS: −UV-C) plasmids in the presence of DIG-dUTP. Incorporation was evaluated during a time course. These pictures are representative of 2 independent experiments.

Plants compensate the deleterious effects of UV radiation by various mechanisms including the accumulation of secondary metabolites [23]. To check whether the UV-C hypersensitive phenotype of *cul4*, *ddb1a* and *ddb2* mutant lines is the consequence of a defect in a DNA repair mechanism, cell extracts of WT, *cul4-1* and *ddb2-2* plants were used in an *in vitro* DNA repair assay as described in [24]. This assay measures the efficiency of DIG dUTP incorporation in a UV-C damaged plasmid in the presence of plant cell extracts. Thereby the efficiency of dark repair of UV-induced DNA damage can be evaluated. Strikingly, cell extracts derived from the mutant plants were less efficient in DIG dUTP incorporation after 1 and 2 h of incubation (Figure 2). Taken together these results show that the UV hypersensitivity of *cul4* and *ddb2* (and most likely *ddb1a*) mutant plants is due to a defect in synthesis-dependent repair of UV-induced DNA lesions, indicating an important role for these factors in the excision repair process.

### Genetic Interactions between Arabidopsis *CEN2*, *RAD1*, *RAD10*, *CUL4*, *DDB1A,* and *DDB2*

**Figure 3:**
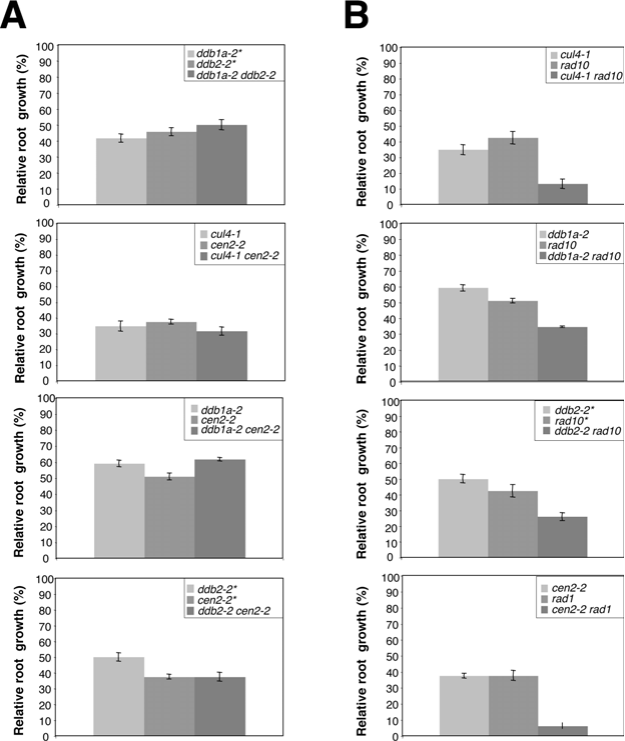
Genetic interactions in NER and HR. One-week-old single and double mutant plants (A) *ddb1a-2-ddb2-2*; *cul4-1-cen2-2*; *ddb1a-2-cen2-2*; *ddb2-2-cen2-2* (B) *cul4-1-rad10*; *ddb1a-2-rad10*; *ddb2-2-rad10*; *cen2-2-rad1* were exposed to 600 J/m^2^ of UV-C. Root growth was measured 24h following irradiation. Root growth was calculated relative to the corresponding untreated plants (±SEM). Eight plants per replicate were used and experiments were triplicated. For all the single and double mutants p<0.05, compared to WT plants. Because *ddb2-2* is in a different Arabidopsis ecotype, the single control mutants were selected as segregants from each double mutant involving *ddb2-2* and are indicated by *.

CENTRIN2 (CEN2) and the heterodimer RAD1-RAD10 (XPF-ERCC1) act in the GGR-NER repair pathway [7]. CEN2 is part of the XPC-RAD23 recognition complex, whereas, RAD1-RAD10 in cooperation with RAD2 are the endonucleases excising the bulky DNA lesions. In Arabidopsis CEN2, RAD1-RAD10 are also part of the GGR-NER repair process [25]. In order to define whether *CUL4*, *DDB1A* and *DDB2* act in the same repair pathway as CEN2 and RAD1-RAD10 different double mutants were produced and characterised for their UV-C sensitivity by using the root growth assay. Double *ddb1a-2 ddb2-*2, *cul4-1 cen2-2*, *ddb1a-2 cen2-2* and *ddb2-2 cen2-2* mutant plants did not exhibit significant additive effects on root growth inhibition compared to the respective single mutants (Figure 3A). These results are consistent with epistatic interactions between *CUL4*, *DDB1A*, *DDB2* and *CEN2* indicating that they act in the same pathway.

The same approach was used using Arabidopsis *RAD1* or *RAD10* defective plants. Contrary to the results observed with *cen2-2,* all double mutant plants (*cul4-1 rad10*, *ddb1a-2 rad10*, *ddb2-2 rad10* and *cen2-2 rad1*) exhibit an enhanced UV-C sensitivity compared to the respective single mutants (Figure 3B). These synergistic genetic interactions suggest that UV induced DNA lesions are processed by at least two different repair pathways in which RAD1 and RAD10 seem to play a role. Although it is assumed that RAD1 and RAD10 are mainly involved in NER, a role in homologous recombination (HR) has also been reported [26],[27]. In addition, UV was already shown to stimulate somatic homologous recombination [28]. Thus our genetic approach suggests that not only NER, but also HR, contributes to maintain genome integrity upon UV irradiation.

### Defect in Photolyase Expression (*PHRI*) Enhances UV-C Sensitivity of *CUL4*, *DDB1A,* and *DDB2*

**Figure 4:**
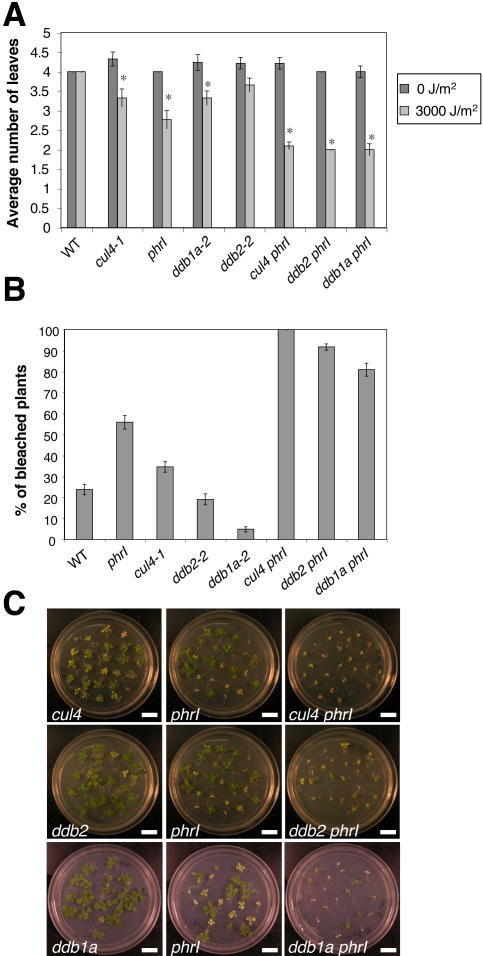
Genetic interactions between CUL4, DDB1A, DDB2 and UVR2. Ten-day-old single (*cul4-1*, *ddb1a-2*, *ddb2-2*, *phrI*) and double mutant plants (*cul4-1 phrI*, *ddb1a-2 phrI*, *ddb2-2 phrI*) were irradiated with UV-C (3000 J/m^2^) 3 times in row every 2 days. (A) Graph representing the average number of leaves. Counting was one week after the last UV-C exposure. At least 20 plants were used per replicate and this experiment was duplicated. T-test *p<0.05, compared to WT plants. (B) Graph representing the percentage of bleached plants. Counting was 2 weeks after the last UV-C exposure. At least 20 plants were used per replicate and this experiment was duplicated. (C) Pictures showing the phenotype of plants 14 days after the last UV-C exposure (Bar = 1 cm).

In contrast to mammals, plants and other organisms have a direct DNA repair mechanism, which involves photolyases [3]. It has previously been shown that the Arabidopsis *UVR2* gene encodes a photolyase (PHR1) specific for CPDs [4],[5], which are the main UV-induced DNA lesions. Because *CUL4*, *DDB1A* and *DDB2* act in the GGR-NER pathway (see above), we checked how a combined defect in both DNA repair pathways affects UV-C tolerance in plants. For this, double *cul4-1 phrI*, *ddb1a-2 phrI* and *ddb2-2 phrI* mutant plants were produced and exposed to chronic UV-C doses. Hence, all double mutants exhibited a significant higher UV sensitivity compared to the corresponding single mutant plants reflected by a reduction in the number of newly formed leaves and a higher number of bleached plants (Figure 4A–C). This synergistic genetic interaction between both pathways indicates that NER and not only photolyase plays a major role to maintain genome integrity in green tissues, which are constantly exposed to sunlight.

### *In vivo* Characterisation of the CUL4-DDB1A-DDB2 Complex

**Figure 5:**
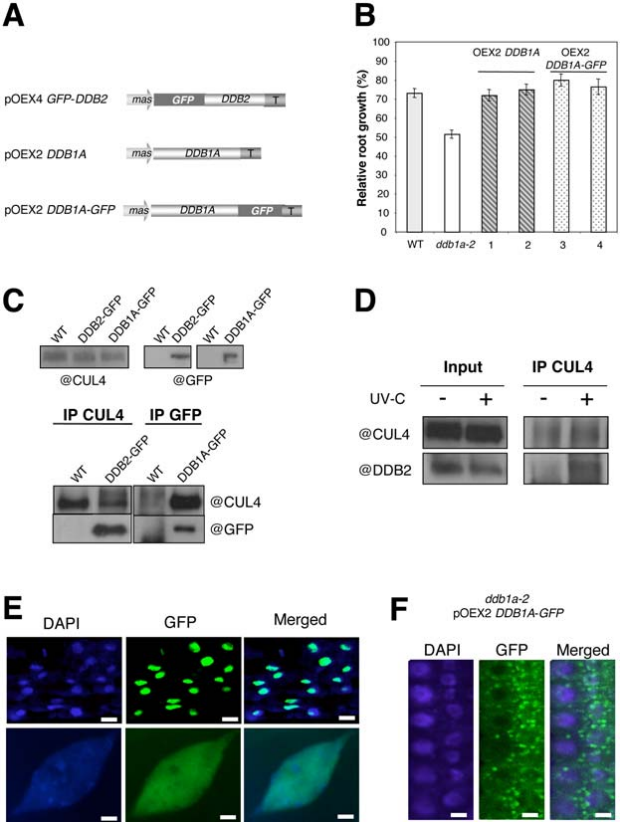
DDB1A and DDB2 interaction with CUL4 and their subcellular localisation. (A) Schematic representation of the constructs (mas: mannopine synthase; GFP: Green Fluorescent Protein; T: nos terminator). (B) Root growth assays showing complementation of *ddb1a-2* with either DDB1A or DDB1A-GFP ectopically expressed proteins. (C) *In vivo* pull down of CUL4 with DDB1A and DDB2 proteins. WT, pOEX4*GFP-DDB2* and *ddb1a-2* pOEX2*DDB1A-GFP* plants were used for immunoprecipitation assays using either anti-CUL4 (Bernhardt et al., 2006) or anti-GFP antibodies. WT plants were used as controls. (D) *In vivo* pull down of CUL4 complex upon UV-C exposure. WT plants were used for immunoprecipitation assays using anti-CUL4 antibody. CUL4 and DDB2 were detected before (−) and 15 min upon UV-C exposure (+). (E) Localisation of GFP-DDB2 fusion protein in root cells using confocal microscopy (Bar = 50 µm) and immunolocalisation in root cells (Bar = 5 µm). (F) Localisation of DDB1A-GFP fusion protein in root cells using confocal microscopy (Bar = 25 µm). All pictures are representative of 3 different experiments using independent transgenic lines. Chromatin is stained by DAPI (blue).

Next, we investigated whether Arabidopsis CUL4, DDB1A and DDB2 proteins form a complex *in planta*. As no antibody against DDB1A was available and because we further aimed to determine the subcellular localisation of DDB1A and DDB2, we first produced Arabidopsis transgenic plants expressing GFP-tagged versions of both proteins (Figure 5A). To test whether the DDB1A-GFP fusion protein was functional, complementation of the UV-C sensitivity of *ddb1a-2* plants was analysed using the root growth assay. Expression of both untagged DDB1A and DDB1A-GFP fusion protein complement the *ddb1a-2* UV-C sensitivity compared to the control plants to the same extent (Figure 5B). Therefore DBB1A-GFP expressing plants are suitable for further analyses. Pair-wise immunoprecipitation experiments were conducted using either anti-CUL4 or anti-GFP antibodies. Indeed we found that both DDB1A and DDB2 co-immunoprecipitate with CUL4 (Figure 5C). In addition, we showed an enrichment of DDB2 when co-immunoprecipitated with CUL4 upon UV-C exposure compared to untreated plants (Figure 5D).

In XP group E patients, a point mutation in the DxR motif (R273H) of the WD40 domain in DDB2 abolishes its interaction with DDB1 leading to the human genetic disorder XPE [16]. This motif is also conserved in Arabidopsis. Therefore, we investigated the interaction between the mutated Arabidopsis DDB2 protein DDB2 (R343H) and DDB1A using the yeast two-hybrid assay. In contrast to human, interaction between DDB1A and DDB2 was not abolished by the DxR mutation (Figure S2). This shows that some features of the Arabidopsis DDB1A-DDB2 interaction may differ from those of human. Overall, our immunoprecipitation and previously published yeast two-hybrid assays [20] suggest that CUL4, DDB1A and DDB2 are part of a same protein complex *in vivo.*

### *In vivo* Localisation of DDB1A and DDB2

To better understand the regulation of the CUL4-DDB1A-DDB2 complex in the context of DNA repair, the subcellular localisation of DDB1A and DDB2 was investigated. Arabidopsis plants expressing GFP-tagged versions of DDB1A and DDB2 were analysed using confocal microscopy. We observed that GFP-DDB2 is localized exclusively in the nucleus (Figure 5E). This result is consistent with the published work in metazoans, including human [15]. To investigate into more details the localisation of DDB2, we performed immunolocalisation experiments using the anti-GFP antibody. We found DDB2 broadly distributed on chromatin with a lower intensity at the chromocenters and nucleoli (Figure 5E). This result is also in agreement with the recent work of [15] showing a homogenous distribution of human DDB2 on chromatin in absence of genotoxic stress. On the other hand, the subcellular localisation of DDB1 in mammalian cells is less well understood and seems more complex [19],[29],[30]. Here we show that Arabidopsis DDB1A-GFP is mainly located in the cytosol and is enriched in speckles of unknown origin (Figure 5F). This subcellular localisation contrasts with the situation in mammals, where both nuclear and cytoplasmic localisations have been reported. The strict cytosolic localisation of plant DDB1A raises the question whether this protein relocates to the nucleus under genotoxic stress conditions in order to contribute to the DNA repair process.

### DDB1A Spatio-Temporal Dynamics upon UV Exposure

**Figure 6:**
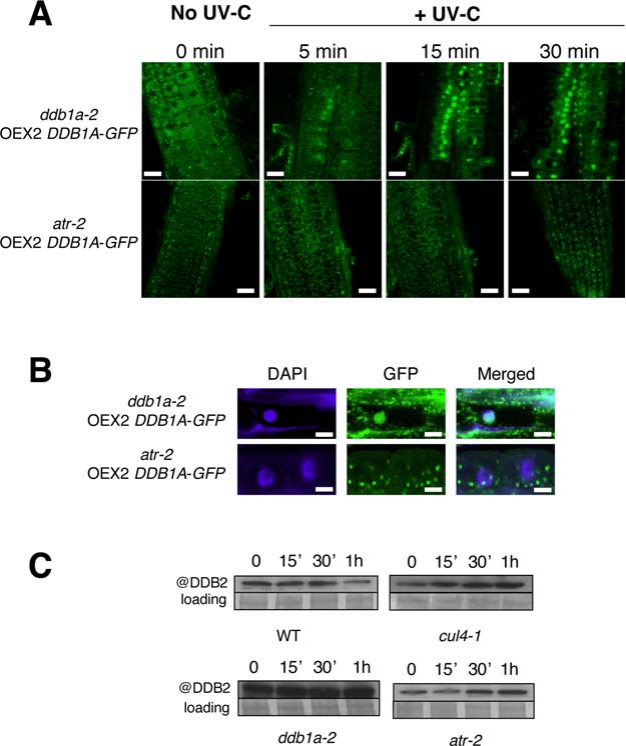
ATR-dependent DDB1A-GFP nuclear shuttling and DDB2 stability. (A) DDB1A-GFP dynamics upon UV-C exposure (600 J/m^2^) in WT (upper panel) and in *atr-2* mutant plant (lower panel). Bar = 75 µm. Pictures are representative of 3 different experiments using 2 independent transgenic lines. (B) Higher magnification of DDB1A-GFP localisation 15 min upon UV-C exposure in either *ddb1a-2* or *atr-2* mutant backgrounds. Chromatin is stained by DAPI (blue). (C) Immunoblot revealing DDB2 content upon UV-C exposure (900 J/m^2^) in WT, *cul4-1*, *ddb1a-2* and *atr-2* Arabidopsis plants. Coomassie blue staining was used as loading control.

To investigate whether DDB1A protein accumulates in the nucleus, at least transiently upon DNA stress, we performed a time course experiment following UV-C exposure. Strikingly, as soon as five minutes upon exposure to UV-C, we observed a clear enrichment of the DDB1A-GFP signal in nuclei of UV-C treated roots and this signal even increased up to 30 min (Figures 6A and 6B). The UV-C dependent nuclear shuttling of DDB1A suggests an upstream level of control of the CUL4-DDB1A-DDB2 complex.

Ataxia telangiectasia-mutated and Rad3-related (ATM, ATR) kinases play a crucial role in sensing and transmitting DNA damage signals to downstream effectors of cell-cycle progression or DNA repair components. The response of ATR to UV lesions and stalled replication forks occurs independently of ATM, although both ATM and ATR have also cooperative roles [31]. Thus we investigated whether DDB1A nuclear shuttling depends on ATR. To do so, we engineered *ATR*-deficient plants (*atr-2*) expressing the DDB1A-GFP construct and monitored the DDB1A dynamics upon UV-C exposure. In contrast to the rapid DDB1A shuttling from the cytoplasm to the nucleus in WT plants, DDB1A-GFP remained excluded from the nucleus in UV-exposed *atr* deficient plants (Figures 6A and 6B). Thus we conclude that both DDB1A and DDB2 accumulate in the nuclei of cells upon UV-stress, most likely to promote excision repair and that this mechanism is dependent on ATR.

### DDB2 Protein Stability Is CUL4- DDB1A- and ATR-Dependent

In order to study the regulation of DDB2 stability in the context of DNA repair, the DDB2 protein content was determined upon exposure of WT, *CUL4*-, *DDB1A-* and *ATR*-deficient plants to UV-C. Both the endogenous DDB2 and the GFP-DDB2-tagged protein steady state levels in WT plants start to decrease between 30 min to 1 hour after UV-C treatment (Figure 6C and Figure S3). Two hours after UV-C treatment neither the DDB2 protein content (Figure S3B) nor the DDB1A subcellular localisation (data not shown) was re-established. As similar decrease in DDB2 protein content was observed when plants where treated with cisplatin (Figure S3A), which also induces bulky DNA lesions that are processed by the excision repair pathway. However, it is noteworthy that the decrease in the steady state level of DDB2 was only evident after 6 hours, most likely because the drug uptake by the plant and its genotoxic effects need more time, compared to UV-treatment. In contrast to UV and cisplatin treatments, no DDB2 decay was observed upon exposure of WT plants to hydrogen peroxide, that induces DNA lesions which are not predominantly repaired by the GGR-NER pathway (Figure S3A).

In human, DDB2 stability is controlled by the CUL4-based E3 ligase [32]. Therefore we tested whether CUL4 and DDB1A are essential components for UV-induced degradation of DDB2 *in vivo* by exposing Arabidopsis *CUL4*- and *DDB1A*-deficient plants to UV-C. Interestingly, DDB2 protein level remained stable and even slightly increased upon UV-C exposure in *cul4-1* and *ddb1a-2* mutant plants, suggesting that DDB2 is a target of the CUL4-DDB1A E3 ligase complex (Figure 6C and Figure S3C). Moreover, all our attempts to express GFP-DDB2 in either *cul4-1* or *ddb1a-2* mutant backgrounds failed leading to plant death, suggesting that DDB2's turnover is crucial for plant survival (data not shown).

As we showed that DDB1A shuttles from the cytoplasm to the nucleus upon UV-C exposure in an ATR-dependent manner (Figures 6A and 6B), we investigated whether a defect in this mechanism abolishes also DDB2 decay. Thus, *atr-2* mutant plants were irradiated with UV-C and DDB2 protein content was determined during a time course. Interestingly, no decay of DDB2 protein was observed (Figure 6C and Figure S3C), indicating a role for ATR in the regulation of DDB2 stability, most likely via the direct or indirect control of DDB1A shuttling to the nucleus.

## Discussion

### A Role of Arabidopsis CUL4-DDB1A-DDB2 in NER

Regulation of protein stability through the ubiquitin proteasome system (UPS) is a major mechanism underlying many cellular and organismal processes, such as cell division, signal transduction in hormonal and developmental pathways, immune defence and DNA repair, among others [33],[34],[35]. Ubiquitylation requires ubiquitin protein-ligases (E3s) that transfer ubiquitin to an internal lysine residue of the target protein. Among the different E3 enzymes, the composition of CUL4-based E3 ligases was only recently identified [36]. CUL4 binds RBX1 to recruit a specific E2 ubiquitin conjugating enzyme and also binds DDB1, an adaptor protein, which itself associates with substrate receptors. Affinity purification of CUL4 E3s from mammalian cells identified various WD40 proteins as possible substrate receptors [37], [38]–[39],[40]. These WD40 proteins interact with DDB1 through a DxR motif, but for most of them, their roles and substrates remain unknown.

Plant genomes contain also numerous WD40 proteins carrying the DxR motif [41], one of them is Arabidopsis DDB2. In human, a point mutation in the DxR motif (R273H) of DDB2 leads to the XPE genetic disorder by abolishing its interaction with DDB1 [8]. Although DDB1 is well conserved in plants and the fact that the DxR motif is present in the Arabidopsis DDB2 homolog, a similar point mutation did not abolish DDB1A-DDB2 interaction, at least in yeast. Therefore, it is possible that some characteristics of DDB1-DDB2 interaction differ in plant. However recombinant and purified human DDB2 (R273H) variant is also able to form a complex with DDB1 *in vitro* [42]. It will now be interesting to check the *in vivo* effects of the plant DDB2 (R273H) mutant. Arabidopsis DDB1A is also part of other protein complexes, which most likely do not contain DDB2. It was reported that DDB1A forms a complex with the ubiquitin-conjugating enzyme variant COP10 and the de-etiolated 1 (DET1) protein to regulate photomorphogenesis [43], possibly via a CUL4-dependent E3 ligase activity [44]. Thus it is likely that the dynamics of the different protein complexes involving CUL4 and DDB1A must be tightly controlled and that DDB1A is more abundant than its various substrate receptors, such as DDB2. It is noteworthy that the Arabidopsis genome encodes also a second DDB1-related protein, called DDB1B sharing 93% protein sequence similarity with DDB1A. However, it is still unknown whether DDB1B plays similar or distinct roles than DDB1A.

Overall, our data point to an involvement of CUL4, DDB1A and DDB2 as effectors of synthesis dependent repair of UV-C induced DNA damage as described in human cells. First, we showed that both Arabidopsis DDB1A and DDB2 co-immunoprecipitate with CUL4 from plant extracts, supporting the existence of a CUL4-DDB1A^DDB2^ complex *in planta*. Second, mutations of *CUL4, DDB1A* and *DDB2* lead to plant hypersensitivity to bulky DNA lesions induced by UV-C or cisplatin. This is in agreement with the recently reported UV hypersensitive phenotype of DDB2-deficient plants [21]. Most importantly, we showed that cell extracts prepared from CUL4 and DDB2 deficient plants are less efficient in dark repair of UV-induced DNA damage. Third, the *cul4-1 cen2-2*, *ddb1a-2 cen2-2* and *ddb2-2 cen2-2* double mutant genetic analysis strongly suggests that *CUL4*, *DDB1A* and *DDB2* act in the same pathway than *CEN2*, which was previously shown to be involved in the NER pathway and to interact with the Arabidopsis XPC homolog, AtRAD4 [22],[45].

### DDB1A Nuclear Shuttling and DDB2 Stability Regulated by the Protein Kinase ATR

In mammals, the recognition step of the NER pathway involves both the CUL4-DDB1-DDB2 and XPC-RAD23-CEN2 complexes [13]. Although DDB1-DDB2 binds DNA helix distortions with the highest affinity, its exact mode of action is still controversial. However, whether DDB2 directly recruits XPC to DNA lesions [13] or only creates a chromatin environment facilitating the assembly of NER components [15], it is believed that DDB2 needs to be degraded in order to allow efficient processing and repair of DNA lesions. In mammals, proteolysis of DDB2 is performed by the 26S proteasome and specifically depends on the CUL4-DDB1 E3 ubiquitin ligase [10], [11]–[12], [13]–[15]. Similarly to mammalian DDB2, the Arabidopsis homolog is also degraded after UV-stress by a mechanism that is CUL4-dependent. It is noteworthy that the plant DDB2 protein decay is only partial and starts as soon as 1 hour or even less after UV stress, which contrasts with the situation reported in some animal cells where DDB2 decay was only detected 2 to 4 hours upon UV exposure [15]. The rapid DDB2 decay in plants compared to animals could be explained by the predominant role of the GGR repair process in cooperation with either direct repair or homologous recombination to maintain efficiently genome stability upon UV irradiation.

At present, little is known about the upstream signals leading to this degradation process and/or the factors controlling specifically the CUL4 E3 ligase activity. It is well described that neddylation/deneddylation is a general and important process to regulate E3 ligases functions [46]. Indeed it was shown that COP9/signalosome regulate CUL4 activity [9]. However more specific mechanisms may also contribute to the control of the E3 ligase complexes. We showed in this study that a functional Arabidopsis DDB1A fluorescent protein localises predominantly in the cytoplasm in the absence of UV irradiation, which was puzzling for a protein supposed to act at the chromatin level. However, upon UV exposure DDB1A relocates rapidly into the nucleus. This observation raised the question of how DDB1A shuttling to the nucleus is controlled in order to contribute to an efficient DNA repair process. Experiments on transfected cells indicate that human DDB1 is also primarily located in the cytoplasm, but shows a dynamic nuclear accumulation induced by UV light [29],[30]. Moreover, when DDB2 was co-expressed together with DDB1, the later protein showed increased accumulation in the nucleus suggesting that DDB2 plays an active role in this translocation process. Interestingly, a recent study showed that the replication checkpoint protein Claspin is required for the turnover of DDB2 [47]. SiRNA-mediated knockdown of Claspin abolishes the degradation of DDB2 upon UV exposure and impairs its co-localization at damaged DNA sites. Claspin is required for activation of the ATR-mediated DNA damage checkpoint response, leading to arrest of DNA replication and inhibition of cell cycle progression [48]. This would suggest that crosstalk exists between cell cycle checkpoint and NER.

ATR plays a central role in cell-cycle regulation, transmitting DNA damage signals to downstream effectors [31]. Therefore the link between ATR signalling and DDB2 stability was investigated. Arabidopsis is a material of choice to study the role of ATR in DNA damage signalling because ATR deficient plants are viable [49] whereas lack of ATR expression is lethal in mammals [50]. Using an Arabidopsis *atr* mutant plant, we showed that in such a genetic background DDB2 was stabilised upon UV exposure, indicating a direct or indirect role for this signalling pathway in the control of the first step of the NER process. In addition, DDB1A shuttling from the cytoplasm to the nucleus upon UV exposure is also ATR-dependent, suggesting that this mechanism might control DDB2 turnover via the assembly of the CUL4-DDB1A E3 ligase in the nucleus. Our results highlight the fact that ATR signalling controls another effector of the excision repair machinery in addition to XPA [51], a protein that is not conserved in plants. A similar shuttling upon UV exposure was already described for the Arabidopsis CEN2, another component of the GGR pathway and for the large subunit of the tobacco ribonucleotide reductase [45],[52]. The control of DDB1A nuclear translocation by the ATR kinase and subsequently DDB2 turnover illustrates a novel and specific level of regulation of Cullin-based E3 ligase complex activity.

### Crosstalk of Different DNA Repair Pathways to Maintain Genome Integrity upon UV Stress

Plants have to take benefit of sunlight for photosynthesis, but at the same time, have also to face UV damages on DNA. Thus to maintain genome integrity, efficient DNA repair processes are needed. In contrast to mammals, plants have a specific DNA repair pathway, the direct DNA repair that is mediated by photolyases. In this pathway, UV-induced pyrimidine dimmers are reversed without DNA excision in the presence of visible light [3]. In Arabidopsis two photolyases, PHR1 [4],[5] and UVR3 [6] are specific for each type of UV induced DNA lesions, the CPDs and 6-4 photoproducts, respectively. It is believed that in green tissues and in presence of light the direct repair is the predominant pathway, whereas in absence of light the NER also called dark repair, is preferentially used. Using a genetic approach we found a synergistic genetic interaction between *CUL4, DDB1A, DDB2* and the photolyase *PHRI* regarding UV sensitivity. This strongly suggests that both pathways contribute together to efficiently repair UV lesions in green tissues and does not restrict the CUL4-DDB1A-DDB2 pathway to DNA repair in non-light exposed tissues (e.g., in roots). Moreover, based on our results (Figure 4), the restriction of the NER pathway to proliferating cells as previously suggested [53] also seems unlikely. In addition CUL4, DDB1A and DDB2 are expressed in various Arabidopsis cell types including post-mitotic cells of mature leaves (unpublished data).

Interestingly, we also identified a synergistic genetic interaction between the components of the NER and RAD1-RAD10. Although the RAD1-RAD10 heterodimer is mainly known to be involved in the removal of bulky DNA lesions during the NER process, it was shown that these enzymes also contribute to the removal of non-homologous overhangs during HR in plants [26],[27]. Moreover in *Saccharomyces cerevisiae*, mutations in RAD1 and in RAD10 reduce intra-chromosomal recombination [54],[55]. The synergistic genetic interactions observed with the Arabidopsis *rad1* and *rad10* mutations stress the point that HR, in addition to NER, is activated to maintain genome integrity upon UV exposure.

**Figure 7:**
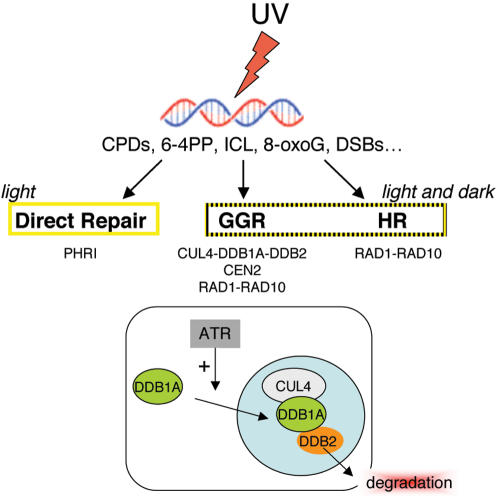
Schematic representation of DNA repair pathways in plant cells and control of DDB2 stability upon UV exposure. As a consequence of UV irradiation, different types of DNA damage are produced such as cyclobutyl pyrimidine dimers (CPDs), (6-4) photoproducts (6-4) Pps, Inter/Intra Cross Link (ICL), 8-oxoG or even DNA double-strand breaks (DSBs). In order to repair these lesions different repair processes including Direct Repair, Homologous Recombination (HR) and Global Genome Repair (GGR) are activated. The GGR pathway involves the CUL4-DDB1A ubiquitin E3 ligase complex. Based on our data, we propose the following model: Under normal growth conditions DDB1A localises predominantly to the cytoplasm, whereas CUL4 and DDB2 are both located into the nucleus. Upon UV irradiation, DDB2 recognises bulky DNA lesions and DDB1A shuttles from the cytoplasm to the nucleus in an ATR-dependent manner (direct or indirect). This allows DDB2 degradation by the 26S proteasome and most likely permits subsequent steps of the excision repair process to occur efficiently.

It is noteworthy that UV radiations induce primarily CPDs and 6-4 photoproducts but also DSBs that need to be efficiently repaired. Moreover, Arabidopsis *PHRI* mutants or plants defective in NER, such as the *Atcen2* mutant [22] exhibit not only hypersensitivity to UV but also enhanced somatic homologous recombination frequency [56]. Taken together our data indicate that at least three different repair pathways (Figure 7), direct repair, NER and HR, cooperatively contribute to maintain genome integrity in different tissues upon exposure to UV. Hence, combined disruption of these pathways becomes dramatic for plant development and survival under UV stress conditions.

## Materials and Methods

### Plant Material and Growth Conditions

Arabidopsis mutant plants have been identified using the web assisted program: http://signal.salk.edu/cgi-bin/tdnaexpress. *ddb1a-1* (SALK 055584), *ddb1a-2* (SALK 038757), *cen2-2* (SM-3-20758), *atr-2* (SALK 032841), *rad10* (SALK 077000), *rad1* [57], *phrI* (WiscDsLox 466C12) are in Columbia ecotype whereas *ddb2-2* (RATM53-3351-1) is in Nossen ecotype. The *cul4-1* mutant is described in [20]. For *in vitro* culture plants were germinated on GM medium (MS salts (Duchefa, The Netherlands), 1% sucrose, 0.8% agar, pH 5.8) in the presence or absence of a selectable agent. Plants were grown in a culture chamber under a 16h/8h photoperiod (22°C/20°C). For soil cultured plants, seeds were sown (20/pot) and put at 4°C in the dark during 3 days. The pots were transferred in greenhouse and kept under a regime of 16h/8h photoperiod (20°C/16°C; 70% humidity). At the 4 leaf developmental stage, plants were individualized and grown for further experiments.

### Molecular Characterisation of Insertion Lines and Production of Transgenic Plants

Single and double homozygous mutant plants were characterised by PCR (see supplemental material for details). The effect of the T-DNA or transposon on gene expression was determined by RT-PCR for *DDB1A* and western blotting for DDB2, respectively. Total RNA from WT plants or insertion lines was prepared using Trizol (GibcoBRL). The reverse transcription was performed using 4.5 µg of total RNAs, hexamers and the Reverse Transcription kit (Applied Biosystems). The PCR reaction was performed in 50 µl reaction mixture containing 1 µl of the RT reaction mixture, 1.25U of ExTaq (Takara), 1.5 mM MgCl_2_, 200 µM of each dNTP and 2 µM of gene specific primers The genes specific primers and PCR conditions are in supplemental material. For all genes different dilutions of the RT reaction mixture and number of PCR cycles were used to confirm that the PCR amplifications were within the linear range.

The pOEX2 and pOEX4 vectors [22] driving expression of DDB1A or DDB2, respectively, under the control of the mannopine synthase constitutive promoter were constructed. The cDNAs of Arabidopsis *DDB1A* and *DDB2* were amplified by PCR from the stock center clones (C104978 and U61992), sequenced and cloned into the pOEX2 and pOEX4 vectors, respectively between the *Nco*I-*Avr*II sites for DDB2 and *PmeI-AvrII* sites for DDB1A. For GFP fusion, the GFP coding sequence was inserted into the *NcoI* sites for pOEX4GFP-DDB2 fusion and *AvrII* site for pOEX2DDB1A-GFP fusion. The resulting plasmids pOEX2DDB1A, pOEX2DDB1A-GFP and pOEX4GFP-DDB2 were mobilized into *Agrobacterium tumefaciens* and used to transform Arabidopsis WT and *ddb1a-2* plants. *DDB1A* expression was verified by RT-PCR and *DDB1A*-*GFP* using primers combination e and f (see supplemental material).

### Treatment of Plants with DNA Damaging Agents

To evaluate the UV-C (254 nm) sensitivity, we used a root-growth assay. Three-day-old *in vitro* germinated WT and homozygous mutant plants were transferred to square plates containing GM medium and grown vertically for an additional day. Root length was measured 24h upon UV-C exposure (600 J/m^2^) using a Stratalinker (Stratagene). The relative root growth was calculated: (root length treated/ root length untreated)×100 (±SEM). Eight plants per replicate were used. Experiments were performed in triplicates. No significant differences in root growth were observed between all untreated mutants and WT plants under our growth conditions and at the time the plant material was analysed.

To evaluate the UV-C (254 nm) sensitivity on whole plants 1-week-old *in vitro* germinated WT and homozygous mutant plants were irradiated with UV-C (3000 J/m^2^) 3 times in row every 2 days using a Stratalinker (Stratagene). Plants were immediately returned to the growth chamber. The bleached phenotype was observed one week later. At least 20 plants per replicate were used and the experiment was duplicated.

For treatment with Cisplatin (Cis) and hydrogen peroxyde (H_2_O_2_), 10 day-old Arabidopsis seedlings were transferred from solid GM plates into 24-well culture plates placed in sterile liquid GM medium containing 0; 0.1; 0.5; 1; 5 and 10 µM of Cisplatin (Sigma) or 0; 1.25; 2.5; 3.75 and 5 mM of H_2_O_2_ (Sigma). The use of different concentrations allowed us to check the linear range of the treatment. The relative weight was calculated: (weight treated/ weight untreated)×100 (±SEM) for the most representative concentration (5 µM Cisplatin and 2.5 mM H_2_O_2_). Eight plants per concentration were used. Experiments were performed in triplicates.

### *In vitro* Repair Assay

The *in vitro* repair assay according to [24] was performed on cell extracts prepared from *cul4-1*, *ddb2-2* Arabidopsis mutant plants and from WT as control. The pGEX and pBSK plasmids were linearised using the *Sma*I restriction enzyme and purified using the Gel extraction kit (Qiagen). The pGEX-linearised plasmid was either UV-C damaged (450 J/m^2^) using the Stratalinker (Stratagene) and used as repair substrate. The non-damaged pBSK-linearised plasmid was used as internal control. Fifteen µg of protein extracts were used per time point and mixed with 200 ng of damaged plasmid (pGEX) and 300 ng of non-damaged plasmid (pBSK). The reaction was stopped 30 min, 1 h or 2 h after incubation by rapid frozen into liquid nitrogen. Plasmids were purified using the Gel extraction kit (Qiagen) and separate by electrophoresis on a 0.8% agarose gel. DNA was transferred to a nylon membrane (Roche) by capillary transfer and DIG detection procedures were performed using the DIG Nucleic Acid Detection kit (Roche) according the manufacturer's instruction.

### Microscopy Analysis and Immunolocalisation

Roots of vertically grown one week-old *in vitro* germinated WT and pOEX4 GFP-DDB2 plants were excised and images were captured as a stacked series using the Axiovert 100M confocal microscope (Zeiss). For immunolocalisation roots were excised, fixed and immunostained using the anti-GFP antibody (Clontech) as described in [58]. Roots of DDB2-GFP plants were fixed during 45 min in 4% formaldehyde prepared in 1×PME (50 mM Pipes, 5 mM MgSO_4_, 5 mM EGTA, pH 6.9) and washed 5 times 5 min in 1×PME. Roots were digested for 45 min in a 0.3% (w/v) cellulase, 0.3% (w/v) pectolyase solution (Onuzuka RS cellulase) prepared in 1×PME and washed 5 times for 5 min with 1×PME. Root tips were squashed onto poly-lysine-coated slides and allowed to air dry. Slides were rehydrated in 1×PME and incubated for 15 min with 0.5% Triton X-100 (Sigma) in 1×PME and rinsed with 1×PME. Slides were incubated 3 times 5 min in 1×PME and immersed in −20°C methanol for 10 min. Slides were rinsed in 1×PBS for 10 min and washed 3 times 5 min in 1×PBS at room temperature, then incubated with the monoclonal anti-GFP antibody (Clontech). Antibody was diluted 1∶750 in 3% BSA, 0.05% Tween-20, 0.02% NaN_3_ in 1×PBS. Hundred µl of diluted primary antibody were applied to each slide for 3 h at room temperature. Slides were washed 3 times 5 min in 1×PBS and incubated for 3 h at room temperature in presence of 100 µl of FITC-conjugated goat anti-mouse secondary antibody diluted in (1∶1000) in 3% BSA, 0.05% Tween-20, 0.02% NaN_3_ in 1×PBS dilution solution. Slides were washed as before and mounted in DAPI-containing mounting medium (Vectashield, Vector). Nuclei were visualized using a Nikon E800 fluorescence microscope.

For monitoring DDB1A dynamics, roots of vertically grown one week-old *in vitro* germinated *ddb1a-2* pOEX2DDB1A-GFP plants were irradiated with UV-C at 600 J/m^2^ using a the Stratalinker (Stratagene) and images were captured every 5 min during the first hour following UV-C exposure.

### Antibodies and Immunoblotting

A peptide of 16 amino acids (DLPSREIVHSNDFNRH) was synthesised and used to immunise rabbit. The antiserum was immunopurified against the peptide according the protocol of BIOMOL.

For determining DDB2 content, 2-week-old germinated WT, *cul4-1* and *atr-2* plants were grown *in vitro* on GM medium. Plants were UV-C irradiated (900 J/m^2^) using the Stratalinker (Stratagene). Eight plants per time point (before irradiation, 15 min, 30 min, 1 h and 2 h after irradiation) were harvested. Total protein was prepared using a denaturating buffer. Twenty µg of total protein were separated by SDS gel and blotted onto Immobilon-P membrane (Millipore). The anti-peptidic anti-AtDDB2 antibody was used to 1/1000 dilution. Quantification of protein content was performed using the Image J software relative to the loading controls.

### List of Primers Used in This Study

a: TTTGGTATGGCTTTTACTTCCTG
b: TGGTCATCTGATATATTGCTTCC
c: TGAGAGACCATAACCATCTTCTAGC
d: GATGTGCAAAGCCCACTATTG
e: GGAAAATGAACCAACTAAGGAAGG
f: TTGATTGATGATTGATTTGACTC
g: GATTCAAGATTGATGATCCATTCCAC
h: AAAATCCTCACGACGTGTCAG
LBa1: TGGTTCACGTAGTGGGCCATCG
LBb1: GCGTGGACCGCTTGCTGCAACT
Ds5: TCCGTTCCGTTTTCGTTTTTTAC
Ds3: CCGGATCGTATCGGTTTTCG

## Supporting Information

Figure S1 UV-C sensitivity of DDB1A and DDB2 overexpressing plants and mutants sensitivities to Cisplatin and H_2_O_2_. (A) Root-growth assay. One-week-old *DDB1A* and *DDB2* independent overexpressor lines and WT control plants were exposed to 900 J/m2 of UV-C. Root growth was measured 24h following irradiation. Eight independent plants were analysed per lines and the experiment was performed in triplicates. (B) One-week-old mutant (*cul4-1*, *ddb1a-2*, *ddb2-2*), and WT control plants were cultured in presence of 5 µM of Cisplatin. Weight was measured one week after. Relative weight was calculated relative to the corresponding untreated plants (±SEM). Eight plants per replicate were used and experiments were triplicated. For all mutants p<0.05, compared to WT plants. (C) One-week-old mutant (*cul4-1*, *ddb1a-2*, *ddb2-2*), and WT control plants were cultured in presence of 2.5 mM of H_2_O_2_. Weight was measured one week after. Relative weight was calculated relative to the corresponding untreated plants (±SEM). Eight plants per replicate were used and experiments were triplicated. For all mutants no significant difference was found compared to WT plants.
(0.63 MB TIF)

Figure S2 DxR-mutated DDB2 binds DDB1A in yeast. (A) Schematic representation of DDB2 carrying four WD40 domains. The DxR motif at the end of the third WD40 domain is indicated as well as the point mutation R343H used for the yeast two-hybrid assay. (B) Yeast two-hybrid interaction between DDB1A and DDB2 and its mutated version (R343H). No auto-activation by the single plasmids was observed when the yeast strains were grown on LTA medium.
(0.78 MB TIF)

Figure S3 Steady state of GFP-DDB2 protein levels and quantification of DDB2 protein levels upon various genotoxic stresses. (A) Immunoblot revealing GFP-DDB2 content of pOEX4*GFP-DDB2* Arabidopsis transgenic plants upon exposure to UV-C (900 J/m^2^), Cisplatin (10 µM) and H_2_O_2_ (5 mM). Coomassie blue staining was used as loading control. (B) Immunoblot revealing DDB2 content in WT plant up to 2 h following UV-C exposure and the quantification of DDB2 protein levels normalised to the loading controls. (C) Graphs representing the quantification of DDB2 protein levels normalised to the loading controls upon exposure to UV-C in WT plants and in the different mutant backgrounds used. Quantifications are representative of 2 independent experiments, one of them is presented in Figure 6C.
(0.82 MB TIF)
