# Supplementary figures and images for: Regulation and Role of Arabidopsis CUL4-DDB1A-DDB2 in Maintaining Genome Integrity upon UV Stress
Source: PLoS Genet. 2008 Jun 13;4(6):e1000093. doi: 10.1371/journal.pgen.1000093 (PMC2396500; doi:10.1371/journal.pgen.1000093)

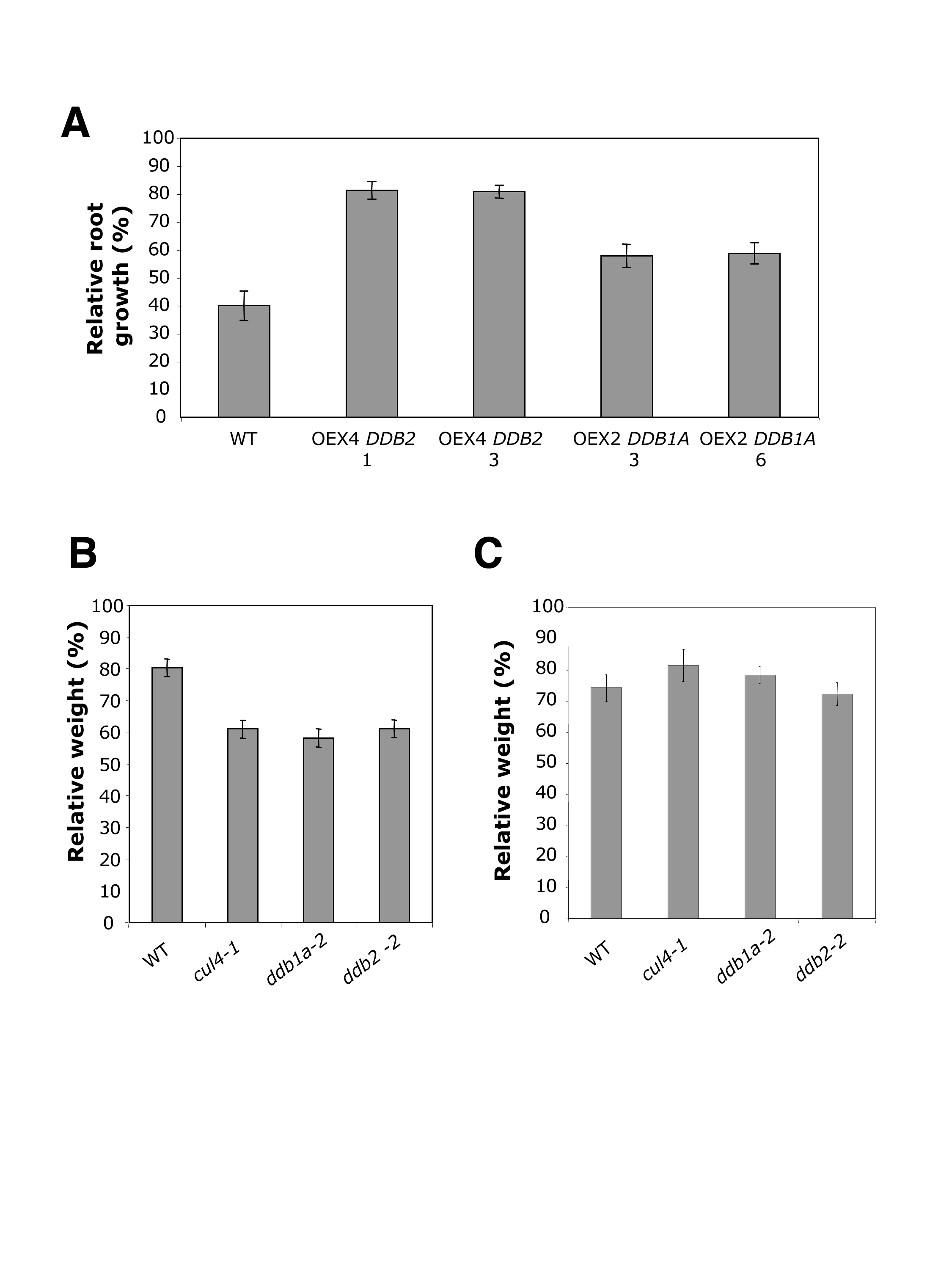

Supplement: Figure S1 — UV-C sensitivity of DDB1A and DDB2 overexpressing plants and mutants sensitivities to Cisplatin and H2O2. (A) Root-growth assay. One-week-old DDB1A and DDB2 independent overexpressor lines and WT control plants were exposed to 900 J/m2 of UV-C. Root growth was measured 24h following irradiation. Eight independent plants were analysed per lines and the experiment was performed in triplicates. (B) One-week-old mutant (cul4-1, ddb1a-2, ddb2-2), and WT control plants were cultured in presence of 5 µM of Cisplatin. Weight was measured one week after. Relative weight was calculated relative to the corresponding untreated plants (±SEM). Eight plants per replicate were used and experiments were triplicated. For all mutants p<0.05, compared to WT plants. (C) One-week-old mutant (cul4-1, ddb1a-2, ddb2-2), and WT control plants were cultured in presence of 2.5 mM of H2O2. Weight was measured one week after. Relative weight was calculated relative to the corresponding untreated plants (±SEM). Eight plants per replicate were used and experiments were triplicated. For all mutants no significant difference was found compared to WT plants. (0.63 MB TIF) [file pgen.1000093.s001.tif]

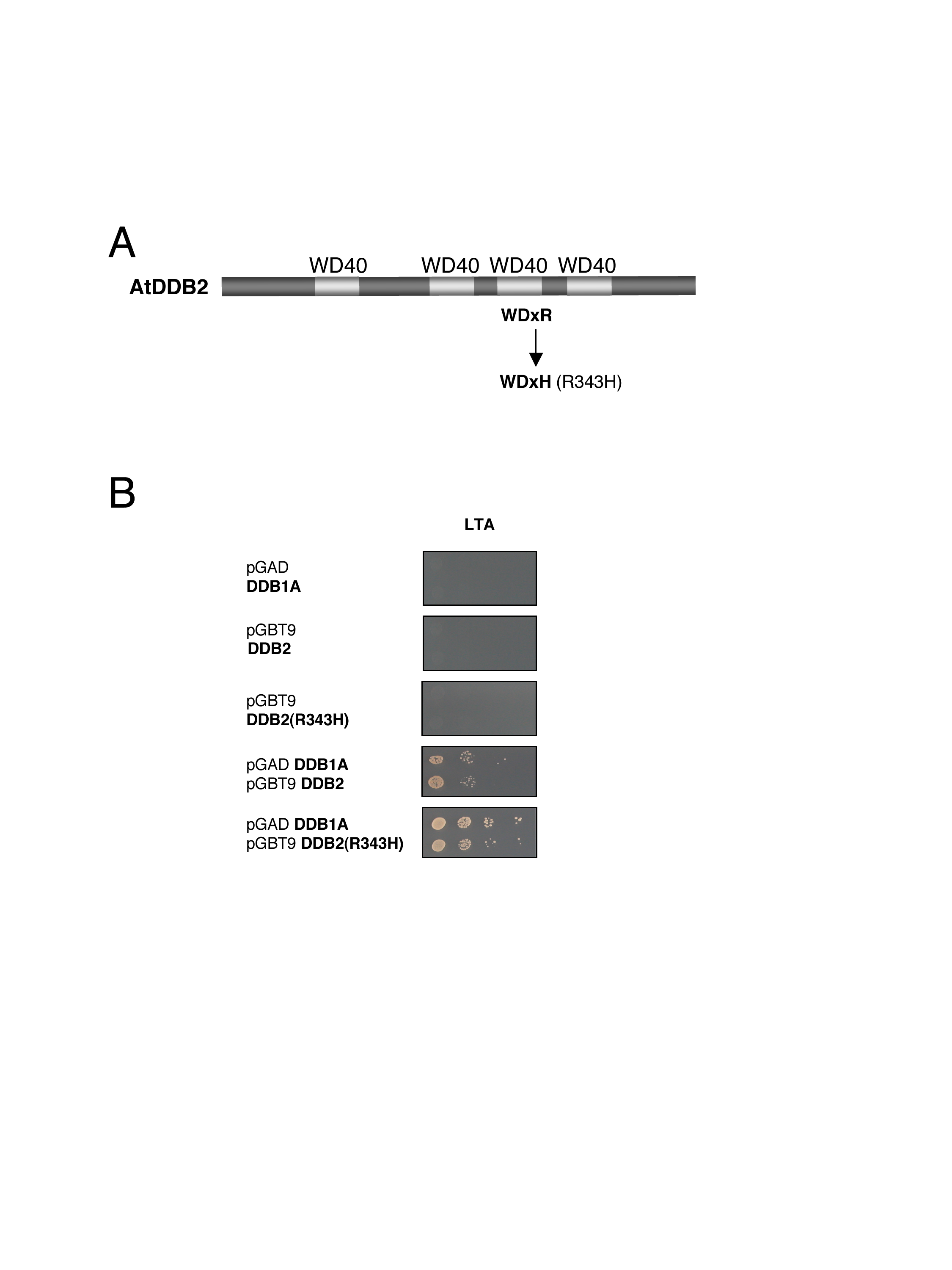

Supplement: Figure S2 — DxR-mutated DDB2 binds DDB1A in yeast. (A) Schematic representation of DDB2 carrying four WD40 domains. The DxR motif at the end of the third WD40 domain is indicated as well as the point mutation R343H used for the yeast two-hybrid assay. (B) Yeast two-hybrid interaction between DDB1A and DDB2 and its mutated version (R343H). No auto-activation by the single plasmids was observed when the yeast strains were grown on LTA medium. (0.78 MB TIF) [file pgen.1000093.s002.tif]

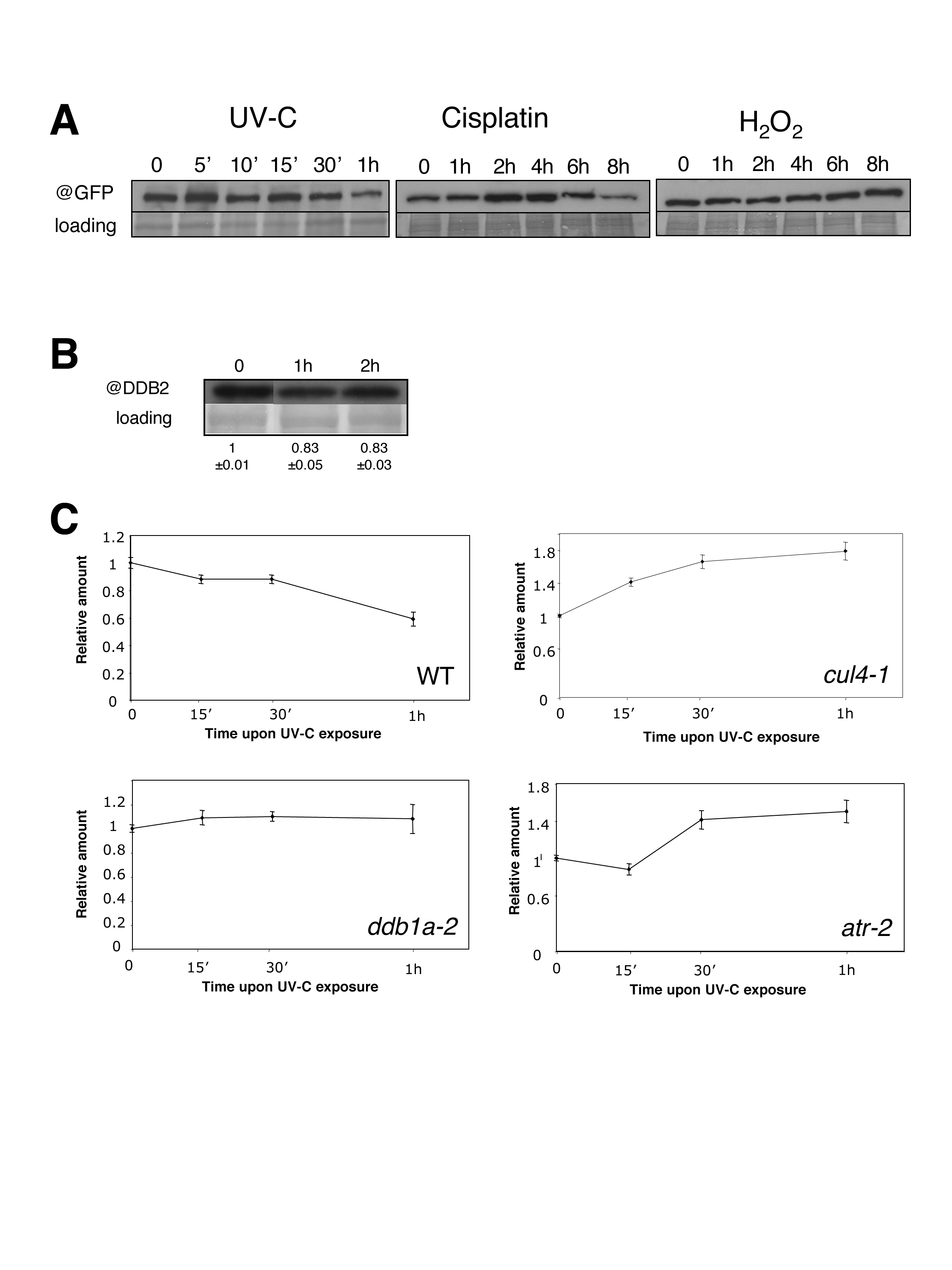

Supplement: Figure S3 — Steady state of GFP-DDB2 protein levels and quantification of DDB2 protein levels upon various genotoxic stresses. (A) Immunoblot revealing GFP-DDB2 content of pOEX4GFP-DDB2 Arabidopsis transgenic plants upon exposure to UV-C (900 J/m2), Cisplatin (10 µM) and H2O2 (5 mM). Coomassie blue staining was used as loading control. (B) Immunoblot revealing DDB2 content in WT plant up to 2 h following UV-C exposure and the quantification of DDB2 protein levels normalised to the loading controls. (C) Graphs representing the quantification of DDB2 protein levels normalised to the loading controls upon exposure to UV-C in WT plants and in the different mutant backgrounds used. Quantifications are representative of 2 independent experiments, one of them is presented in Figure 6C. (0.82 MB TIF) [file pgen.1000093.s003.tif]
